# From Talk to Text: Improving Feedback Concordance With a Simple Intervention

**DOI:** 10.1002/aet2.70181

**Published:** 2026-05-20

**Authors:** Rachael Tesorero, Isabel J. Hsu, Beth Holman, Joseph House, Dan Dinh, Laura R. Hopson

**Affiliations:** ^1^ Department of Emergency Medicine University of Michigan Ann Arbor Michigan USA; ^2^ Department of Internal Medicine University of Michigan Ann Arbor Michigan USA; ^3^ University of Michigan Medical School Ann Arbor Michigan USA; ^4^ Department of Emergency Medicine and Pediatrics University of Michigan Medical School Ann Arbor Michigan USA; ^5^ Department of Anesthesiology University of California Los Angeles‐Harbor California USA; ^6^ Department of Emergency Medicine University of Michigan Medical School Ann Arbor Michigan USA

## Abstract

**Background:**

High‐quality written feedback is critical to learner development. Actionable feedback is essential in emergency medicine (EM), where students do not consistently rotate with the same supervisor. Medical students frequently report dissatisfaction with feedback due to inadequate quantity, vagueness, and discordance between verbal and written elements. Educational scripts, sometimes referred to as “scaffolds”, prompt people to respond in a desired manner and show potential as an intervention to encourage clinical feedback.

**Methods:**

We incorporated a “stop and think” verification question in medical student assessments. Supervisors answered yes or no to the statement: “I discussed this feedback with the student.” We hypothesized this would improve students' assessment of feedback quality and verbal‐written feedback concordance. Outcomes include three feedback perception questions added to the end‐of‐clerkship student evaluations and qualitative analysis of the types of narrative feedback provided. We prospectively collected 8 months of assessment data (*n* = 82) and compared pre‐intervention data to 5 months of post‐intervention data (*n* = 49) for length and content.

**Results:**

The percentage of students rating the quality of clerkship feedback as excellent or very good remained stable (62% vs. 74%, *p* 0.76). Students reporting concordance of the verbal and written feedback the majority of the time improved significantly from 72% to 87% (*p* < 0.001). Importantly, the proportion of students reporting feedback concordance more than 75% of the time increased from 37% to 67%. The content and type of written feedback did not change. The intervention did not change assessment response rate for supervisors or learners (36% vs. 36%; 74% vs. 72%, respectively).

**Conclusions:**

A verification question added to student assessment forms can positively impact student perceptions of verbal‐written feedback concordance without altering the content of feedback provided, including actionability. This easy‐to‐implement strategy provides one mechanism to encourage supervisors to provide more feedback to trainees.

## 1 Background

Feedback is critical to learning, and necessary to the learner at all stages of development [1, 2, 3]. Providing specific and timely feedback is especially difficult in the emergency department (ED) setting, where shift‐based work makes consistent long‐term interactions less likely [4]. Nationwide trends toward ED overcrowding further exacerbate the challenge of making time to reflect and give feedback [5].

Research in medical trainees reflects the importance of feedback and a desire for higher‐quality feedback on clinical skills [6, 7, 8]. In particular, students express frustration with the tension between the overlap of formative feedback, which is constructive, and summative feedback, which is evaluative and factored into grading [9]. In addition to verbal in‐person feedback, written feedback also provides value to learners [10]. However, the greatest effectiveness derives from multiple modalities of feedback, especially when written and verbal feedback convey concordant information [11, 12].

Medical schools continue to invest in faculty professional development in service of improving feedback for learners [13, 14]. The quantity of students receiving feedback has increased through mechanisms that encourage instantaneous feedback, such as filling out a card in person [15, 16]. Numerous evidence‐based models to optimize improving feedback are propagated at workshops across health professions schools [17, 18, 19, 20, 21]. While effective, these effortful methods require the development and retention of trained faculty, which may limit their impact [22].

Educational scripts, sometimes referred to as “scaffolds”, prompt individuals to respond in desired ways [23]. Scaffolds may be internal or external. External scaffolds, such as expected standards or norms, can influence internal processes and support long‐term behavioral change [23]. This prompting strategy overlaps with classic behavioral psychology in which stimuli or cues are trained to elicit a specific response such as with Pavlovian conditioning [23, 24]. Cues, scaffolds, and social norms are used in public health campaigns [25, 26, 27] and in electronic medical records [27, 28, 29] to promote positive behaviors.

We investigated whether introducing an external scaffold via a single verification question on student assessment forms would improve students' perceptions of concordance between written and verbal feedback—an issue previously identified as a source of frustration among medical students. We also assessed whether this intervention influenced the content of written feedback.

## 2 Methods

We conducted this study at a single large academic medical center in the United States with a required EM experience during the final 16‐month phase of medical school. A prospective sample of rotating students (*n* = 180) was surveyed for nine months before and five months after the intervention, February to September 2023 and October 2023 to March 2024, respectively. They answered questions about feedback quality and concordance embedded in end‐of‐rotation evaluations. Our outcomes used three standard questions after pilot testing with stakeholders. Students rotated at one of three clinical sites affiliated with the medical school.

We analyzed data from supervising attending (*n* = 616 pre‐, 379 post‐intervention) and resident (*n* = 397 pre‐, 301 post‐intervention) physician assessments to analyze for changes in evaluation completion rates, actionability of feedback, comment length, or comment content. To avoid confounders, data collection and analysis was concluded in March 2024 due to the implementation of multiple other medical school interventions for improving the evaluation process. Despite early termination, our a priori calculations assured us of adequate power to determine a 20% difference between the groups for our primary outcome (*n* = 272). This project was reviewed by the IRB and given exempt status.

### 2.1 Intervention

We added a radio button to the existing medical student evaluation forms completed by faculty and residents. The associated question asked whether evaluators discussed the written feedback with the student previously (Figure S1). We also included three questions about the quality and perceived concordance of feedback to the end‐of‐clerkship evaluation forms for medical students (Figure S2). All faculty and resident preceptors were educated as to the attestation button and expectations through email and faculty meeting or residency conference announcements prior to the start of the intervention. Our primary outcomes were the student ratings of feedback quality and concordance pre‐ and post‐intervention. As a secondary outcome, we analyzed evaluation completion rates, written feedback length, and content themes to determine if these changed. We did not expect a change in evaluation completion rates but wanted to ensure our intervention was not an unintentional deterrent.

### 2.2 Analysis

#### 2.2.1 Statistical Analysis

Basic information was maintained in Microsoft Excel (Office 365), which was also used for descriptive statistics. Due to the non‐parametric nature of the dataset, pre‐ and post‐intervention student ratings of the feedback were compared via Mann–Whitney *U* tests. Statistical calculations were performed with SPSS Statistics for Windows v.29 (IBM, Armonk, NY).

#### 2.2.2 Qualitative Analysis

Written comments on student assessment forms were coded into 5 educational domains: Patient Care/Medical Knowledge (PC/MK), Communication (Comm), Professionalism (Prof), Practice‐Based Learning Improvement (PBLI), Teamwork/Leadership/Interprofessional (Team) in accordance with the medical school's assessment domains (Figure S3). We defined a comment to be “Actionable” according to Pavlic et al. [30] “Actionable comments were those that were specific enough that the resident could conceivably choose to change behavior to act upon the comment.”

Two raters (RT and LRH) independently reviewed a random sample of 100 comments (approximately 5% of total comments analyzed). They met and discussed any discrepancies in coding to standardize their approach. They identified significant overlap and difficulty discriminating between PC and MK, so those fields were combined into one domain. After independently coding an additional 25 comments, review demonstrated improved interrater reliability (> 90%). The remaining comments were divided in half, both the before and after, and coded independently by the two raters given the strong interrater reliability.

The frequency of content areas, actionability, and length was compared pre‐ and post‐intervention using Mann–Whitney *U* tests as our coded dataset was non‐parametric. Statistical calculations were performed with SPSS Statistics for Windows v.29 (IBM, Armonk, NY).

## 3 Results

### 3.1 Evaluation of Quality

**FIGURE 1:**
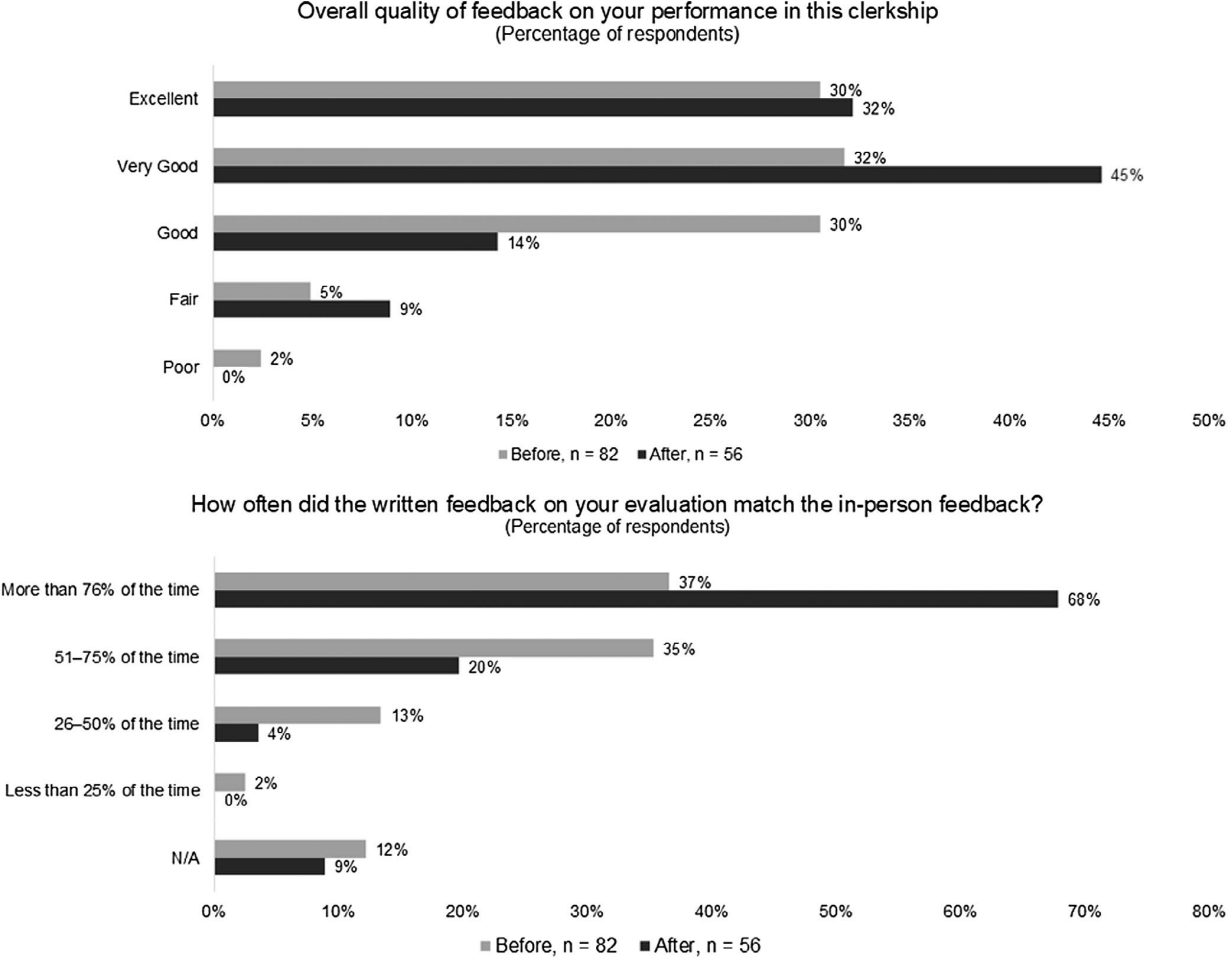
Medical student feedback perception. Students reported increased verbal‐written feedback concordance without a significant difference in feedback quality.

Based on the end of clerkship evaluations, a larger percentage of students indicated they received “Very Good” or “Excellent” quality feedback in the post‐intervention group (62% pre‐ vs. 78% post‐intervention). However, overall there was no statistically significant difference in the quality of the feedback received before versus after adding the feedback indicator (*ρ* = 0.61, Figure 1). The student survey response rate on the end‐of‐rotation evaluation was equivalent in both phases (74% vs. 72%, N.S.).

### 3.2 Evaluation of Concordance

There was a significant improvement in the perception of concordance of the verbal feedback received with the feedback written in the evaluation (*ρ* < 0.001, Figure 1). The percentage of students indicating their written and verbal feedback matched 76%–100% of the time increased from 37% pre‐intervention to 68% post‐intervention.

### 3.3 Evaluation of Feedback Content

The length of the written comments on student assessments did not change pre‐ vs. post‐intervention (mean 71, SD 56 both periods, N.S.). The assessment comments were also found to be similarly actionable both pre‐ and post‐intervention (63% vs. 64%, respectively, *p* = 0.754). There was no significant difference in the specific domains in which students received feedback (Figure S4). There was no change in assessment response rates by supervisors before vs. after the intervention (64% vs. 64%, N.S.).

## 4 Discussion

Our study implemented a single external scaffolding question to written summative evaluations for medical students on the emergency medicine (EM) clerkship. By including a mandatory radio button indicating whether feedback was previously discussed, we significantly increased the proportion of students reporting verbal‐written concordance in their feedback. Importantly, this occurred without seeing degradation in the quality, type, or quantity of feedback received.

Prior work demonstrates that trainees find both formative and summative feedback essential to learning, and that agreement between these two mechanisms is most helpful for professional development [9, 11, 12]. Verbal feedback is especially impactful in EM education, where rapid decision‐making and clinical skills are critical. In high‐pressure environments like EDs, timely feedback allows medical students to refine their skills and build confidence. Providing feedback on procedural skills, patient assessments, and communication in the ED has been shown to improve students' competence and preparedness for real‐world clinical challenges [31, 32]. Written feedback is an important part of the entirety of this feedback process.

External scaffolds and social norms, grounded in psychological theories such as Script Theory and Theory of Planned Behavior along with Social Learning Theory, are particularly effective in fostering a feedback culture in EM [23, 27, 33]. Scaffolds can prompt supervisors to provide consistent, actionable feedback which over time can change the expected social norm for feedback delivery in the ED. For instance, when faculty model feedback‐giving behaviors during high‐stakes situations—such as trauma resuscitations or acute care debriefings—it sets a standard for students to follow. This intervention may also have broader benefits, as social learning theory suggests that students are more likely to learn optimal feedback components when they see faculty actively demonstrating desired behaviors [33]. Furthermore, introducing an external scaffold through a regular feedback prompt, like the question added to our assessment form, along with reminding faculty about feedback during clinical encounters or team briefings, helps normalize the practice of giving feedback despite the hectic nature of emergency care [23, 34]. Notably, these prompts work without requiring a significant amount of extra effort from faculty supporting easy implementation of behavioral change [27]. By embedding feedback into the daily rhythm of ED activities, faculty can create an environment where feedback is an expected, valued component of clinical training, reinforcing a culture of continuous improvement for both students and faculty [27, 32, 34].

## 5 Conclusions

Given concordance between verbal and written feedback improved during this study, our verification question has become permanently integrated into the evaluation forms for the EM clerkship students as well as throughout the medical school's assessments. Through applying a simple external scaffold embedded within an assessment, medical educators can improve the concordance and hopefully effectiveness of feedback delivered to learners across multiple modalities.

## 6 Limitations

Our study is a focused application of a behavioral tool. This intervention was only implemented in one department at a large academic medical center. This study also relies on voluntary completion of post‐clerkship evaluations and student self‐reports, which have inherent biases, such as how much a student subjectively enjoyed the rotation and how much they agreed with the feedback. Additionally, concurrent changes to feedback systems at the medical school level limited the post‐intervention data collection phase. With our sample size, we are unable to determine whether there were temporal relationships to the intervention such as an adjustment period or extinction phenomenon of the frequency of verbal feedback delivery and concordance with written comments.

## Author Contributions

**Dan Dinh:** resources, writing – review and editing. **Isabel J. Hsu:** writing – original draft, writing – review and editing, visualization, resources. **Joseph House:** investigation, writing – review and editing. **Laura R. Hopson:** conceptualization, investigation, writing – original draft, methodology, validation, writing – review and editing, formal analysis, data curation, supervision, resources. **Rachael Tesorero:** conceptualization, investigation, writing – original draft, methodology, validation, writing – review and editing, formal analysis, project administration, data curation, supervision, resources. **Beth Holman:** methodology, writing – original draft, writing – review and editing, investigation, formal analysis, data curation.

## Disclosure

ChatGPT was used to help edit paragraphs and craft the title of the manuscript. AI was not used in any part of the initial draft, data collection, analysis, or conclusions for this study.

## Conflicts of Interest

The authors declare no conflicts of interest.

## Supporting information


**Figure S1:** View of supervisor evaluation form of medical students.
**Figure S2:** Medical student end‐of‐clerkship evaluation form.
**Figure S3:** Medical student core competencies.
**Figure S4:** Evaluation of written feedback.

## Data Availability

The data that support the findings of this study are available from the corresponding author upon reasonable request.
