# Supplementary material for: From Talk to Text: Improving Feedback Concordance With a Simple Intervention
Source: AEM Educ Train. 2026 May 20;10(3):e70181. doi: 10.1002/aet2.70181 (PMC13240450; doi:10.1002/aet2.70181)
Supplement: Supplementary file 1 — Figure S1: View of supervisor evaluation form of medical students. Figure S2: Medical student end‐of‐clerkship evaluation form. Figure S3: Medical student core competencies. Figure S4: Evaluation of written feedback. [file AET2-10-e70181-s001.docx]

**Supplemental Materials:**


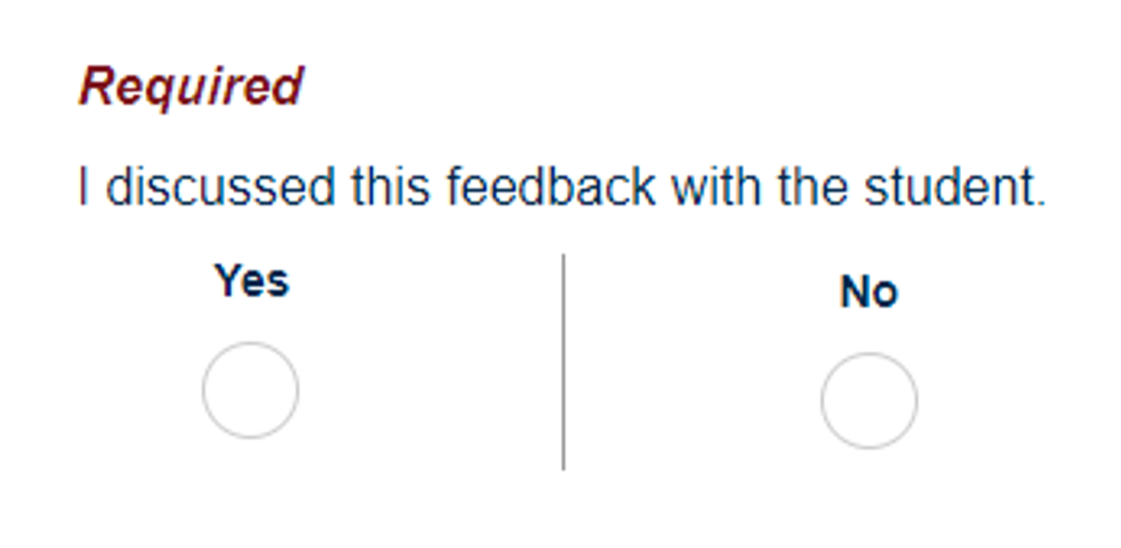


**Supplemental Figure 1**.

View of supervisor evaluation form of medical students.

A required radio button was added to existing summative evaluation forms.

“How would you rate the overall quality of feedback on this rotation?”

Poor

Fair

Good

Very Good

Excellent

“Independent of the quality, how often did the written feedback on your evaluation match the in-person feedback? If you did not receive in-person feedback, select NA.”

NA

0–25% of the time

26–50% of the time

51–75% of the time

76–100% of the time

“Is there anything additional you would like to share with us about your feedback experience on this rotation?”

Free textbox, not required

**Supplemental Figure 2.**

Medical student end-of-clerkship evaluation form

Three survey questions were added to the medical student end of clerkship evaluation form about the quality and concordance of feedback they received during their rotation.


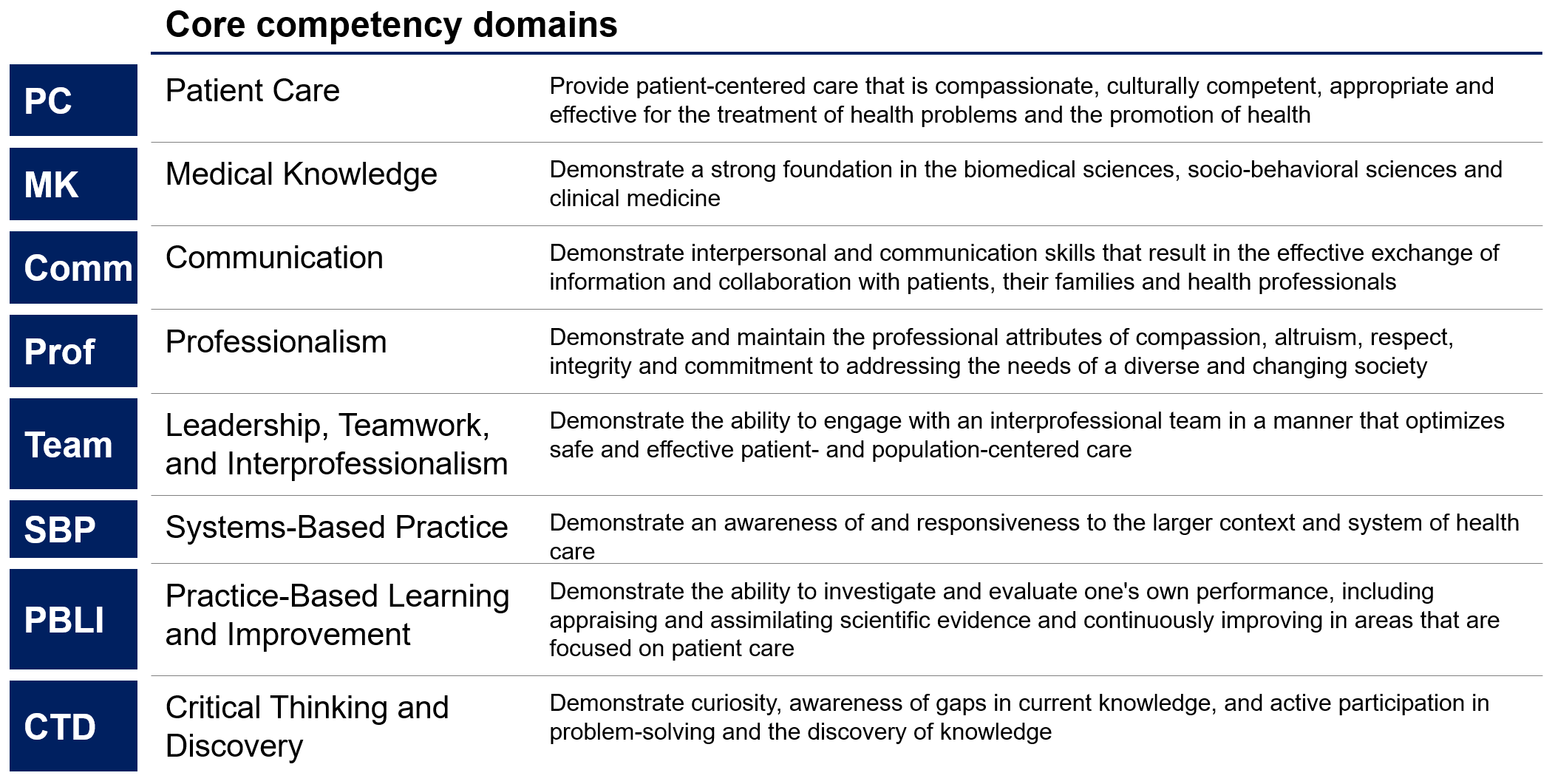


**Supplemental Figure 3.** Medical student core competencies.

Six domains were used to code the written feedback. Patient Care and Medical Knowledge were combined to improve IRR given significant overlap (PC/MK). The domains of Systems-Based Practice and Critical Thinking and Discovery were not evaluated.

|  | Pre-intervention | | Post-intervention | | *P*-value |
| --- | --- | --- | --- | --- | --- |
| Domain | n | % | n | % |  |
| Actionable | 643 | 63% | 434 | 64% | 0.752 |
| PC / MK | 766 | 75% | 518 | 76% | 0.568 |
| Comm | 597 | 59% | 414 | 61% | 0.301 |
| Prof | 435 | 43% | 289 | 43% | 0.964 |
| PBLI | 276 | 27% | 159 | 23% | 0.108 |
| Team | 496 | 49% | 380 | 56% | 0.003 |
| Total | 1,018 | 100% | 679 | 100% |  |

**Supplemental Figure 4.** Evaluation of Written Feedback

The proportion of written feedback addressing Patient Care/Medical Knowledge (PC/MK), Communication (Comm), Professionalism (Prof), and Practice-Based Learning Improvement (PBLI), Teamwork / Leadership / Interprofessional (Team) did not change significantly after the radio button was implemented.
